# Amplified sinus-P-wave analysis predicts outcomes of cryoballoon ablation in patients with persistent and long-standing persistent atrial fibrillation: A multicentre study

**DOI:** 10.3389/fcvm.2023.1110165

**Published:** 2023-03-27

**Authors:** Antonio Creta, Sandrine Venier, Konstantinos Tampakis, Rui Providencia, Juno Sunny, Pascal Defaye, Mark J. Earley, Malcolm Finlay, Ross J. Hunter, Pier D. Lambiase, Nikolaos Papageorgiou, Richard J. Schilling, Simon Sporton, George Andrikopoulos, Elodie Deschamps, Jean-Paul Albenque, Christèle Cardin, Nicolas Combes, Stéphane Combes, Xavier Vinolas, Zoraida Moreno-Weidmann, Taiyuan Huang, Martin Eichenlaub, Björn Müller-Edenborn, Thomas Arentz, Amir S. Jadidi, Serge Boveda

**Affiliations:** ^1^ Barts Heart Centre, St Bartholomew’s Hospital, London, United Kingdom; ^2^ Institute of Health Informatics, University College London, London, United Kingdom; ^3^ Department of Cardiology, Grenoble University Hospital and Grenoble Alpes University, Grenoble, France; ^4^ Electrophysiology & Pacing Department, Henry Dunant Hospital Center, Athens, Greece; ^5^ Département de Rythmologie, Clinique Pasteur, Toulose, France; ^6^ Arrhythmia Unit, Department of Cardiology, Hospital Universitario Sant Pau, Barcelona, Spain; ^7^ Department of Cardiology and Angiology, Medical Center, Faculty of Medicine, University of Freiburg, Freiburg im Breisgau, Germany

**Keywords:** atrial fibrillation ablation outcomes, atrial cardiomyopathy, cryoballoon, P-wave duration, arrhythmogenic substrate, fibrosis, interatrial block, low voltage

## Abstract

**Introduction:**

Outcomes of catheter ablation for non-paroxysmal atrial fibrillation (AF) remain suboptimal. Non-invasive stratification of patients based on the presence of atrial cardiomyopathy (ACM) could allow to identify the best responders to pulmonary vein isolation (PVI).

**Methods:**

Observational multicentre retrospective study in patients undergoing cryoballoon-PVI for non-paroxysmal AF. The duration of amplified P-wave (APW) was measured from a digitally recorded 12-lead electrocardiogram during the procedure. If patients were in AF, direct-current cardioversion was performed to allow APW measurement in sinus rhythm. An APW cut-off of 150 ms was used to identify patients with significant ACM. We assessed freedom from arrhythmia recurrence at long-term follow-up in patients with APW ≥ 150 ms vs. APW < 150 ms.

**Results:**

We included 295 patients (mean age 62.3 ± 10.6), of whom 193 (65.4%) suffered from persistent AF and the remaining 102 (34.6%) from long-standing persistent AF. One-hundred-forty-two patients (50.2%) experienced arrhythmia recurrence during a mean follow-up of 793 ± 604 days. Patients with APW ≥ 150 ms had a significantly higher recurrence rate post ablation compared to those with APW < 150 ms (57.0% vs. 41.6%; log-rank *p* < 0.001). On a multivariable Cox-regression analysis, APW≥150 ms was the only independent predictor of arrhythmia recurrence post ablation (HR 2.03 CI_95%_ 1.28–3.21; *p* = 0.002).

**Conclusion:**

APW duration predicts arrhythmia recurrence post cryoballoon-PVI in persistent and long-standing persistent AF. An APW cut-off of 150 ms allows to identify patients with significant ACM who have worse outcomes post PVI. Analysis of APW represents an easy, non-invasive and highly reproducible diagnostic tool which allows to identify patients who are the most likely to benefit from PVI-only approach.

## Introduction

Catheter ablation represents the most effective treatment for AF, but long-term outcomes in persistent AF are suboptimal, with AF recurring in up to 50% of patients within 12–18 months (1). Low-voltage substrate (LVS) within the left atrium (LA) occurs in atrial cardiomyopathy (ACM) (2–4) and has emerged in the last decade as a relevant arrhythmogenic contributor and possible ablation target in patients with AF (2–9). Several small studies have suggested improved outcomes in persistent AF when LVS was targeted in addition to PVI (2, 5–8). On the other hand, patients with persistent AF and preserved LA voltage are more likely to benefit from PVI-only (3, 10). Non-invasive stratification of patients based on the expected degree of LA fibrosis could allow to identify the best responders to PVI.

Local slow conduction occurs in areas with extensive LVS, causing a prolongation of the total LA activation time and amplified sinus-P-wave (APW) measured from a digitally recorded/amplified 12-lead electrocardiogram (ECG) (3, 10). As such, a prolonged APW enables accurate identification of patients with significant LVS and predicted the increased risk of AF recurrence following PVI in small single-centre studies (3, 10, 11). The aim of the current multicentre study was to investigate the role of APW duration in predicting outcomes of cryoballoon-PVI for non-paroxysmal AF.

## Methods

### Study design

In this observational retrospective study, patients undergoing PVI were included in four tertiary ablation centres in France, the UK and Greece (Centre Hospitalier Universitaire de Grenoble, Grenoble; Clinique Pasteur, Toulouse; Barts Heart Centre, London; Henry Dunant Hospital Center, Athens). Inclusion criteria were age >18 years, persistent (1–12 months of continuous AF) or long-persistent AF (>12 months of continuous AF) and first PVI using cryoballoon-ablation only. Exclusion criteria were prior left-atrial ablations, prior cardiac surgery or adjunctive left-atrial ablations at the index procedure. The endpoint was survival free of any atrial arrhythmia (AF or atrial tachycardia) during follow-up. The study complied with the Declaration of Helsinki and the research protocol was approved by the local review boards.

### Catheter ablation

Procedures were performed under sedation or general anaesthesia, according to each institution's protocol. Venous access was obtained *via* the femoral vein, with use of vascular ultrasound at operator's discretion. A single transseptal puncture was performed under fluoroscopic guidance. Transoesophageal echocardiography was used based on operator preference. All procedures were performed on uninterrupted anticoagulation (therapeutic warfarin or direct oral anticoagulant). Patients received intravenous heparin to maintain an activated clotting time of 300–350 s. Details of the cryoballoon-PVI technique and peri-procedural management at our institutions have been published previously (12, 13). A Medtronic Artic Front Advance™ 28 mm cryoballoon system was adopted for all cases. PVI assessed by entrance and exit block was the procedural endpoint.

### Amplified sinus-P-wave analysis from digital 12-lead electrocardiogram

**Figure 1:**
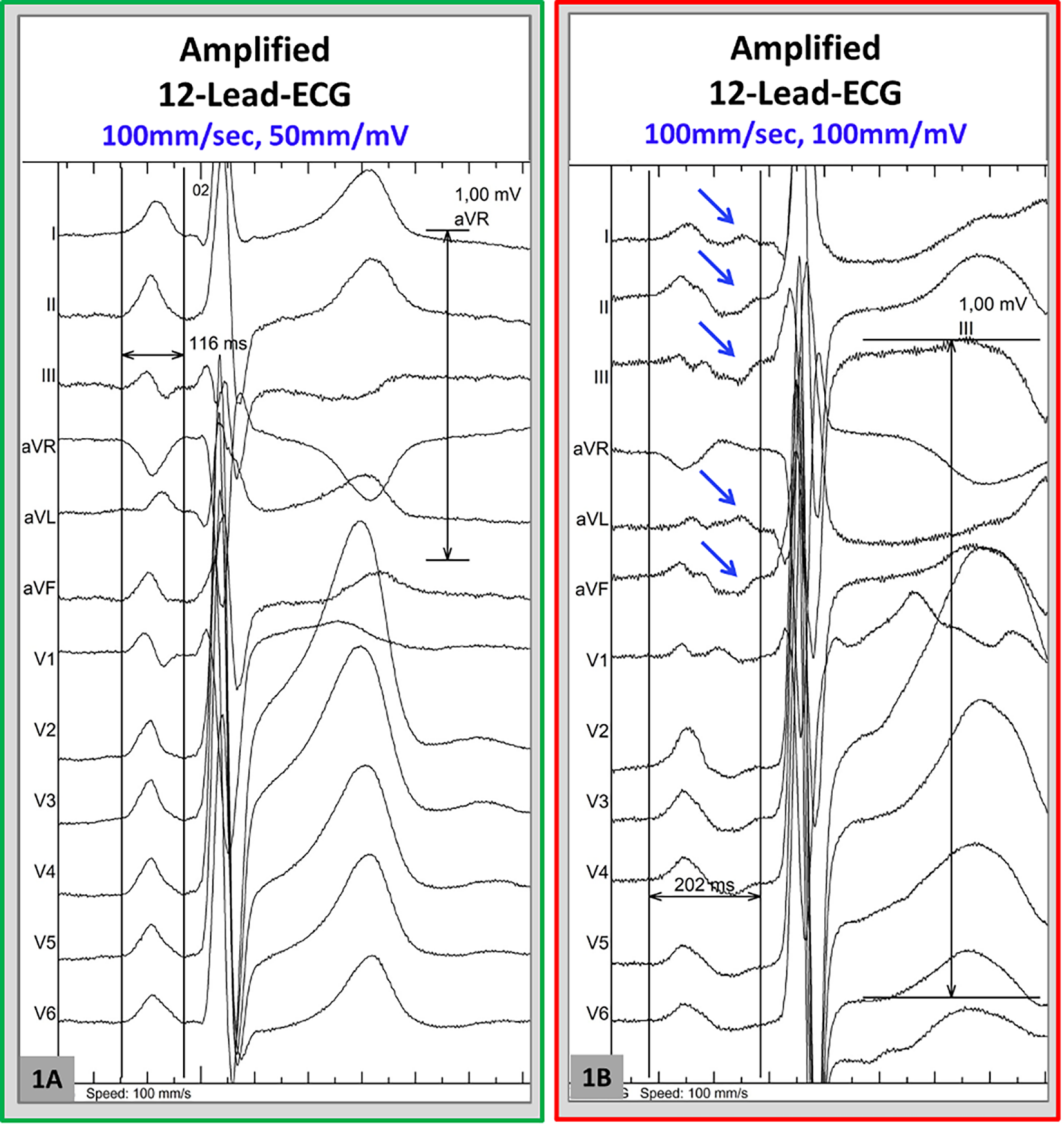
Examples onf amplified-P wave (APW) measurement. 12-lead-ECG in sinus rhythm was recorded digitally at 1,000 Hz sample rate and amplified to 100–200 mm/s sweep speed and 50–100 mm/mV, in order to allow visualization of low voltage portions of the *p*-wave. (**A**) This illustrates an example of APW measurement in a patient without pathological *p*-wave prolongation (total *p*-duration = 116 ms). (**B**) Measurement of the APW reveals a prolonged P-wave duration of 202 ms in this patient.In this example, the late portions of the *p*-wave are best visualized in the leads I, aVL, II, II, aVF (blue arrows).

A 12-lead ECG with electrodes in standard positions was recorded digitally in all patients during sinus rhythm on the day of the procedure using BARD LabsystemPro (Boston Scientific, Marlborough, MA, United States) at 1 kHz sample rate with 0.05 Hz high-pass and 100 Hz low-pass filter settings. If patients were in AF at the beginning of the procedure, direct-current electrical cardioversion was performed in order to allow APW measurement in sinus rhythm. No additional noise filtering/notch filtering was applied to the surface ECG signals. Raw data were then digitally post-processed and amplified to 40–100 mm/mV with 100–200 mm/s sweep speed. APW duration was defined as the interval (ms) between the earliest P wave onset to the latest activation (latest return to the isoelectric line) in any lead. Examples of APW measurement are shown in Figure 1. Patients were stratified based on APW duration using the cut-off of 150 ms which was validated in previous studies (3, 10, 11). An APW duration ≥150 ms was adopted to identify patients with *significant ACM*, based on previous evidence (10). In addition, a morphological analysis of APW was performed to identify the presence of interatrial block or late-terminal P wave, which have been previously shown to correlate with LA LVS. APW-analysis was performed by experienced on-site cardiologists and reassessed by a second cardiologist in an external ECG core laboratory. All of them were blinded to the individual patient's medical history and expected outcome.

### Outcomes and follow-up

The main efficacy endpoint was freedom from any symptomatic or asymptomatic atrial arrhythmia (AF or atrial tachycardia) lasting >30 s following a blanking period of 3 months. Patients were evaluated at 3- and 12- months' post procedure, with further follow-up and testing allowed in case of symptoms. Information collected during follow-up included ECG, and/or 24-hour or 48-hour ECG Holter monitoring and/or implantable cardiac rhythm device check at each visit, according to each institution protocol. Antiarrhythmic drugs post procedure were prescribed at the operator's discretion.

### Statistical analysis

The *χ*^2^ test was used for categorical data and Student's *t*-test for comparison of means was used for comparison of continuous variables. Levene's test was used to check the homogeneity of variance; equivalent non-parametric tests were used when Kolmogorov–Smirnov was in favour of the absence of normal distribution. Kaplan-Meier curves were traced for illustrating freedom from arrhythmia recurrence based on the APW duration (cut-off 150 ms, based on previous studies) (10, 11), and the log rank P test was used for assessing existing differences. Independent predictors of sinus rhythm maintenance after ablation procedure were assessed through Cox regression (Method: Forward Likelihood Ratio, Probability for Stepwise 0.05). Covariates with *p* < 0.10 in the univariable analysis were included in the multivariable model. Results with *p *< 0.05 were regarded as significant. SPSS version 27.0 was used for statistical analysis.

## Results

We included 295 patients (mean age 62.3 ± 10.6, 29.2% female). One-hundred-ninety-three patients (65.4%) suffered from persistent AF and the remaining 102 (34.6%) from long-standing persistent AF. Baseline population characteristics are reported in Table 1. The mean LA diameter and left ventricular ejection fraction were 42.1 ± 7.3 mm and 54.5 ± 10.6%, respectively. The mean APW duration was 154 ± 24 ms (range 90.0–326.0 ms). Significant ACM (i.e., APW duration ≥150 ms) was identified in 168 of 295 patients (57%). Notably, significant ACM was more frequent in patients with a clinical phenotype of persistent AF compared to those with long-standing persistent AF (63% vs. 46%, *p* = 0.006; Table 1). There was no significant difference in use of antiarrhythmic drugs pre and post ablation in patients with APW≥150 ms vs. APW < 150 ms (*p* = 0.56 and *p* = 0.41, respectively). The APW duration did not differ between patients who were on or off antiarrhythmic drugs at the time of procedure (154.1 ± 21.0 vs. 155.2 ± 28.4, respectively; *p* = 0.75). Patients with APW ≥ 150 ms were older compared to those with APW < 150 ms (*p* < 0.001) and had lower GFR (68.8 ± 16.8 vs. 76.6 ± 17.3, *p* = 0.03), but did not differ in other pertinent clinical variables. The large majority of patients were followed-up with serial ECGs and ECG Holter; 11 patients (3.8%) had cardiac rhythm implantable device with arrhythmia detection and storage capacity.

**Table 1:** Baseline characteristics of the study population.

	Total sample (*n* = 295)	APW < 150 ms (*n* = 127)	APW≥ 150 ms (*n* = 168)	*P*
Age (years)	62.3 ± 10.6	60.0 ± 11.9	64.0 ± 9.1	**0**.**002**
Women	86 (29.2%)	42 (33.1%)	44 (26.2%)	0.20
Long-standing persistent AF	102 (34.6%)	55 (53.9%)	47 (46.1%)	**0**.**006**
Congestive heart failure	66 (22.3%)	24 (18.9%)	42 (25.0%)	0.24
Hypertension	139 (47.1%)	56 (44.1%)	83 (49.4%)	0.37
Diabetes mellitus	31 (10.5%)	11 (8.7%)	20 (11.9%)	0.37
History of stroke or TIA	25 (8.5%)	12 (9.4%)	13 (7.7%)	0.60
Vascular disease	28 (9.5%)	14 (11.0%)	14 (8.3%)	0.43
HCM	14 (4.7%)	3 (2.4%)	11 (6.5%)	0.09
Valvular Heart Disease	21 (7.1%)	10 (7.9%)	11 (6.5%)	0.67
Obstructive Sleep apnoea	33 (11.1%)	11 (8.7%)	22 (13.1%)	0.24
CHA_2_DS_2_-VASc	1.8 ± 1.4	1.7 ± 1.5	1.9 ± 1.3	0.12
BMI (Kg/m^2^)	28.5 ± 6.0	28.1 ± 7.2	28.8 ± 5.0	0.41
eGFR (ml/min)	70.9 ± 17.2	76.6 ± 17.3	68.8 ± 16.8	**0**.**03**
LA diameter	42.1 ± 7.3	41.3 ± 7.9	42.3 ± 7.3	0.22
LVEF (%)	54.5 ± 10.6	55.2 ± 11.2	54.1 ± 10.2	0.47
Procedure Duration (min)	80.2 ± 33.3	79.3 ± 33.3	80.8 ± 33.5	0.70
Fluoroscopy Duration (min)	10.8 ± 9.0	10.6 ± 8.8	10.9 ± 9.1	0.80
Class I or III AADs at discharge	214 (72.3%)	90 (70.9%)	124 (73.8%)	0.56

APW, amplified P wave; AF, atrial fibrillation; TIA, transient ischaemic attack; HCM, hypertrophic cardiomyopathy; BMI, body mass index; LA, left atrium; LVEF, left ventricular ejection fraction; AADs, anti-arrhythmic drugs.

### Outcomes post ablation

**Figure 2:**
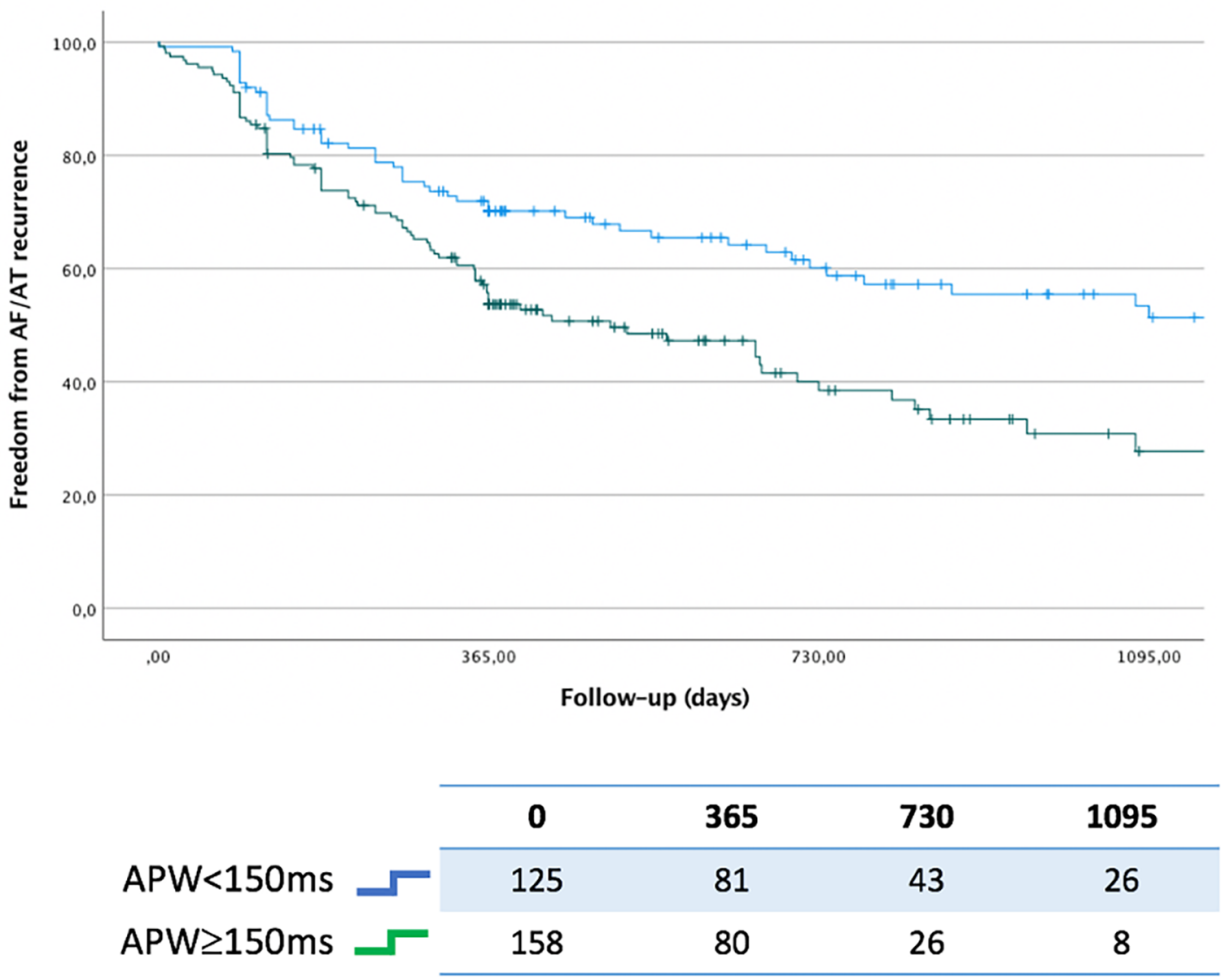
Kaplan-Meier for freedom from atrial arrhythmia relapse stratified by APW duration (APW ≥ 150 ms vs. APW < 150 ms).

**Figure 3:**
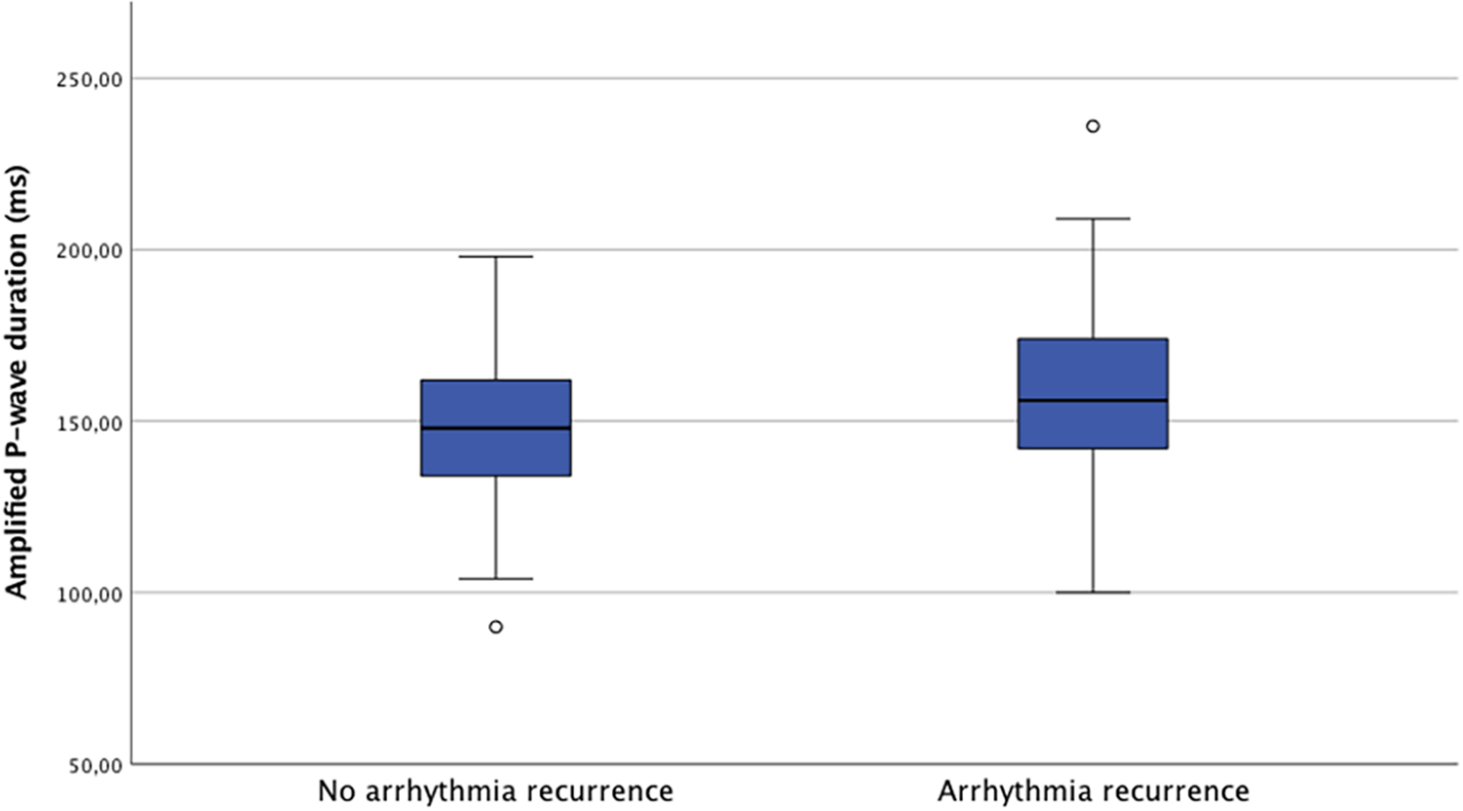
Distribution of APW duration stratified by atrial arrhythmia relapse.

A total of 142 patients (50.2%) experienced arrhythmia recurrence during a mean follow-up of 793 ± 604 days. Twelve patients (4.1%) were lost during follow-up. Patients with APW≥150 ms had a significantly higher recurrence rate post ablation compared to those with APW < 150 ms (57.0% vs. 41.6%, respectively; log-rank *p* < 0.001—Figure 2). The mean APW duration was significantly higher in patients who suffered from arrhythmia recurrence compared to those who did not (149.7 ± 19.0 ms vs. 158.7 ± 27.2 ms, *p* = 0.001). Distribution of APW duration based on outcomes post PVI is shown in Figure 3. On a ROC analysis, APW duration demonstrated an acceptable predictive value for arrhythmia recurrence (AUC 0.60, CI, 0.53–0.66; *p* = 0.03; Youden's index 151 ms; sensitivity 61%, specificity 57.4%).

**Figure 4:**
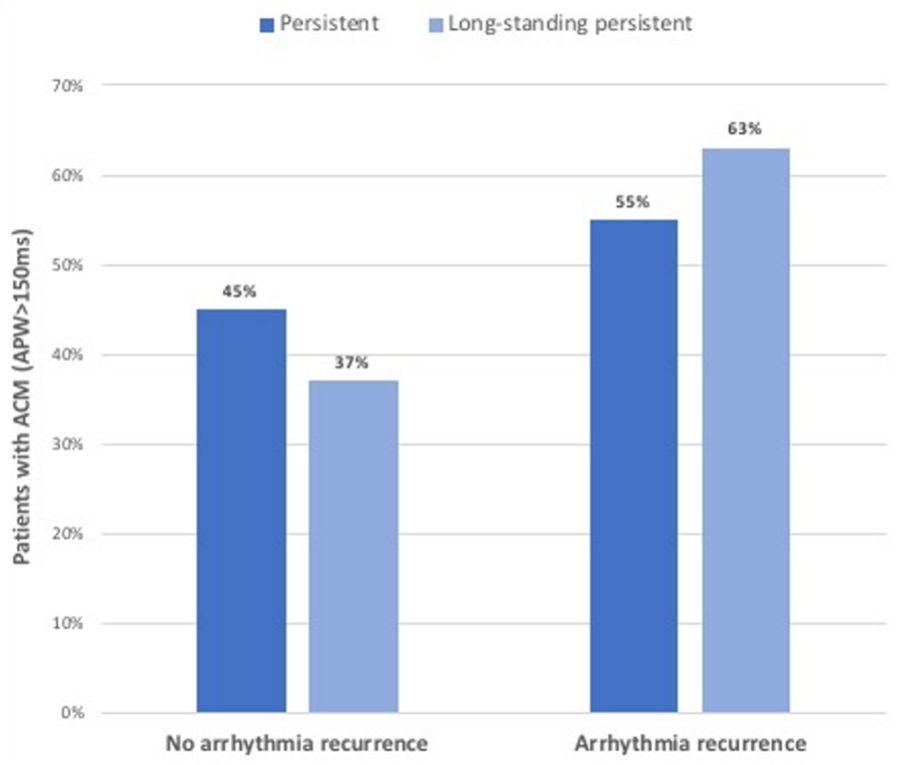
Outcomes of cryoballoon pulmonary vein isolation in patients with atrial cardiomyopathy (i.e. APW ≥ 150 ms), stratified by type of atrial fibrillation (persistent vs. long-standing persistent).

Among patients with APW ≥ 150 ms, the rate of arrhythmia recurrence was 55% and 63% in those with persistent vs. long-standing persistent AF, respectively. These results are shown in Figure 4.

Assessment of independent predictors of arrhythmia relapse is illustrated in Table 2. LA diameter, gender and APW ≥ 150 ms were predictors of recurrence on univariable Cox regression analysis. On multivariable analysis, only APW ≥ 150 ms remained an independent predictor of arrhythmia recurrence post ablation (HR 2.03 CI_95%_ 1.28–3.21; *p* = 0.002).

**Table 2:** Predictors of post-blanking atrial arrhythmia relapse.

Variable	Univariable Cox Regression			Multivariable Cox Regression		
	**HR**	**95%CI**	***P***	**HR**	**95%CI**	***P***
Age (per year)	1.01	0.99–1.03	0.11	–	–	–
Female gender	**1.38**	**0.97**–**1.98**	**0.07**	–	–	–
Long-standing persistent AF	0.96	0.68–1.35	0.82	–	–	–
Congestive heart failure	1.06	0.72–1.58	0.76	–	–	–
Hypertension	1.07	0.77–1.49	0.67	–	–	–
Diabetes mellitus	0.72	0.39–1.32	0.29	–	–	–
Vascular disease	0.80	0.44–1.45	0.46	–	–	–
Obstructive Sleep Apnea	1.24	0.76–2.00	0.38	–	–	–
CHA_2_DS_2_-VASc	1.09	0.97–1.22	0.13	–	–	–
BMI (per Kg/m^2^)	1.00	0.99–1.04	0.99			
eGFR (per ml/min)	1.00	0.98–1.02	0.68	–	–	–
LA diameter	**1.02**	**1.00**–**1.05**	**0.08**	–	–	–
LVEF (per %)	1.00	0.99–1.02	0.46	–	–	–
APW≥150 ms	**1.83**	**1.29**–**2.50**	**0.01**	**2.03**	**1.28**–**3.21**	**0.002**
HCM	0.98	0.48–2.01	0.96	–	–	–

HR, hazard ratio; CI, confidence interval; AF, atrial fibrillation; CHA_2_DS_2_-VASc, cardiac failure or dysfunction, hypertension, age ≥75 years [doubled], diabetes, stroke [doubled] - vascular disease, age 65–74 years, sex category [female]; BMI, body mass index; LA, left atrium; LVEF, left ventricular ejection fraction; APW, amplified P wave; HCM, hypertrophic cardiomyopathy.

#### Subgroup analysis of APW morphology

A combined analysis of APW morphology and duration was conducted in a subgroup of 162 patients. Interatrial block or late-terminal P wave were identified in 38 patients (23.5%) and all but 3 had an APW ≥ 150 ms. Among all patients with APW ≥ 150 ms, incidence of arrhythmia recurrence was slightly higher in those with concomitant interatrial block or late-terminal P waves but this difference was not statistically significant (64.3% vs. 59.2%, *p* = 0.64).

## Discussion

The main finding of the present study is that analysis of APW-duration is effective in predicting long-term success rate of cryoballoon-PVI for non-paroxysmal AF. Patients with significant ACM (i.e., APW duration ≥150 ms), which represent approximately half of this study's cohort, have a significantly higher rate of arrhythmia recurrence post ablation compared to those with APW < 150 ms at a long-term follow-up.

There is a growing body of evidence showing a proarrhythmogenic role of LA fibrosis, which results from the activation and proliferation of cardiac fibroblasts in response to cytokines, hormones and stress factors (14). Fibrotic remodelling of the atria can favour AF onset and maintenance by several mechanisms, including changes in the Ca2^+^ handling and promotion of re-entry (15). LVS defined on electroanatomic mapping has been shown to identify patients with atrial slow conduction substrate who are at increased risk for arrhythmia recurrence post PVI (5–7). Furthermore, targeting LVS may improve long-term efficacy of catheter ablation. Three independent non-randomised clinical studies by Rolf et al. (7), Jadidi et al. (6) and Yamagouchi et al. (5) have shown that patients with persistent AF receiving PVI + LVS ablation had a significantly higher freedom from arrhythmia recurrence compared to PVI-only strategy. These results were also confirmed by a recently published randomised controlled trial by Huo et al. (16). Previous research has demonstrated the high diagnostic performance of APW in identifying patients with ACM presenting LA LVS in endocardial voltage mapping. In a study including 72 patients with persistent AF, Jadidi et al. (10) found that APW duration highly correlated with the extent of LVS measured with electroanatomic bipolar mapping; APW duration ≥150 ms was found to have a 94.3% sensitivity and 91.7% specificity in identifying presence of LVS. Furthermore, APW duration was an independent predictor of the one-year success rate following radiofrequency PVI, with an APW ≥ 150 ms identifying subjects at higher risk of arrhythmia recurrence. In a recent study by Moreno-Weidmann et al. (11), APW duration was an independent predictor of recurrence post radiofrequency ablation for paroxysmal and persistent AF. Similar findings were observed in small single-centre experiences by Liu et al. (17) and Ohguchi et al. (18). Of note, none of the previous studies have explored the predictive value of APW following cryoballoon ablation.

The pattern of progression of LA LVS and related APW modification have been recently described by Müller-Edenborn et al. (3) in a study including 95 patients with persistent AF undergoing high-density activation and voltage mapping in sinus rhythm. The authors found that LVS initially develops in the anteroseptal LA area and leads to a conduction slowing in the Bachmann's bundle, with subsequent APW prolongation. Increasing anteroseptal LVS determines a further conduction delay at the Bachmann's bundle level and results in inter-atrial block, which manifests into a biphasic positive (right atrial)-negative (left atrial) APW morphology in the inferior leads (due to the superiorly directed activation wavefront from the coronary sinus up to the posterior LA wall). Extensive LVS including the LA roof and posterior wall causes a marked prolongation of the APW-duration. Furthermore, in a group of patients with extensive and widespread LA fibrosis, the authors demonstrated the presence of positive deflections (i.e., late-terminal P waves) in the leads I, aVL, V2-V6 resulting from the latest activation of the best-preserved areas with higher voltage at the level of the LA appendage and lateral LA wall. The authors proposed a three-step algorithm based on the analysis of both APW duration and morphology in order to improve patients’ stratification with regards to PVI outcome. In our cohort, the large majority of patients with either a biphasic APW in the inferior leads or late-terminal P wave had also an APW ≥ 150 ms. Rate of arrhythmia recurrence was not significantly higher in patients with concomitant APW ≥ 150 ms and APW morphological changes, although the small sample size of this subgroup should be taken into account. Further studies with large sample size should clarify whether concomitant analysis of P-wave duration and morphology may improve risk stratification.

In keeping with the above observations, the present study confirms in a large and multicentre cohort that APW represents a valuable tool to predict long-term efficacy of PVI. To the best of our knowledge, this is the first study to investigate the prognostic role of APW in patients undergoing cryoballoon-PVI. We believe that this represents a strength of this study because outcomes of cryoballoon-PVI have been proved to be more reproducible and less operator-dependent compared to PVI using radiofrequency techniques (19). The multicentre design of the present study demonstrates the high reproducibility of APW analysis. In addition, with a mean follow-up 793 ± 604 days, this is the first study to confirm that APW analysis can predict PVI outcomes after the first-year post ablation.

Importantly, our cohort included a significant proportion of patients with long-standing persistent AF (i.e., duration >12 months), who were excluded in previous studies on this topic. Long-standing persistent AF was not a significant predictor of arrhythmia recurrence in our multivariable regression model. This finding is particularly relevant as it highlights how the conventional distinction between persistent and long-standing persistent AF may sometimes have a poor correlation with ablation outcomes. This is in agreement with a recent meta-analysis by Clarnette et al. (20). Based on our results, we suggest that a classification of AF based on the degree of LA slow conduction remodelling would be more useful in the clinical setting and the analysis of APW may represent an easy, non-invasive and accurate instrument for this purpose. Cardiac MRI is an alternative method to quantify LA fibrosis (21), however its widespread use is limited by costs, time, necessity of intravenous injection of magnetic resonance contrast agents, as well as need of dedicated sequence/fibrosis detection software, and a trained staff that is currently unavailable at most hospitals.

Finally, an important implication of our data is that the analysis of the APW helps sub-phenotyping persistent and long-persistent AF patients and identifying a subgroup of patients who do well with PVI alone, and a second group of patients whose AF and stage of LA remodelling does not respond as well to PVI. It is therefore clear that PVI-alone may not be enough to patients with APW ≥ 150 ms and it is possible that such group may derive benefit from additional therapy, either with novel drugs or with additional ablation beyond PVI, for example targeting LVS or other non-pulmonary vein triggers. Therefore, routine use of radiofrequency rather than cryoballoon technique may be advocated in this extensive low-voltage sub-phenotype, as the former allows a more extensive and tailored approach. However, this hypothesis should be further investigated in randomised controlled trials. Given that the majority of patients with non-paroxysmal AF are in AF when arrive in the electrophysiology laboratory, physicians may consider performing a direct-current cardioversion before or at the beginning of the procedure in order to measure the APW in sinus rhythm and decide on the ablation strategy.

The main limitation of the present study is its retrospective design which inevitably increases the risk of bias. Thus, our results should be confirmed in prospective studies. We cannot rule out that a proportion of eligible patients were excluded due to impossibility of cardioverting to sinus rhythm and hence measuring the APW; this may have led to a selection bias. Furthermore, this was a multicentre study including experienced large volume centres and may not represent the type of ablation activity performed in other centres with lower caseloads. However, cryoballoon ablation is a very reproducible technique with limited inter-operator differences in terms of efficacy (19). Detection of arrhythmia recurrence was mainly based on a combination of serial ECG and ECG Holter during follow-up, as only a minority of patients had implantable cardiac rhythm device such as loop recorders or pacemakers. As such, we are unable to provide data on AF burden. LA size was assessed using diameter only as no data on LA volume or area were available. Intra-observer reproducibility of APW analysis was not formally assessed. However, in our experience APW measurement is easy and highly reproducible and this also in keeping with previous literature (3, 10, 11). Finally, electrical cardioversion may have caused acute changes of the APW duration and therefore influenced APW measurements in patients requiring cardioversion on the day of the procedure. However, such changes are likely to be minor (22).

## Conclusions

The current multicentre study demonstrates that analysis of amplified p-wave duration in digital 12-lead-ECG (APW) in sinus rhythm predicts arrhythmia recurrence post PVI-only cryoballoon ablation in non-paroxysmal AF. Analysis of APW represents an easy, non-invasive and highly reproducible diagnostic tool which allows to identify patients who are the most likely to benefit from a PVI-only first-intention ablation strategy.

## Data Availability

The datasets presented in this article are not readily available because Data available on request due to privacy/ethical restrictions. Requests to access the datasets should be directed to Corresponding author.

## References

[1] Verma A Jiang CY Betts TR Chen J Deisenhofer I Mantovan R Approaches to catheter ablation for persistent atrial fibrillation. N Engl J Med. (2015) 372:1812–22. 10.1056/NEJMoa1408288 25946280

[2] Kircher S Arya A Altmann D Rolf S Bollmann A Sommer P Individually tailored vs. standardized substrate modification during radiofrequency catheter ablation for atrial fibrillation: a randomized study. Europace. (2018) 20:1766–75. 10.1093/europace/eux310 29177475

[3] Müller-Edenborn B Chen J Allgeier J Didenko M Moreno-Weidmann Z Neumann FJ Amplified sinus-P-wave reveals localization and extent of left atrial low-voltage substrate: implications for arrhythmia freedom following pulmonary vein isolation. Europace. (2020) 22:240–9. 10.1093/europace/euz297 31782781

[4] Goette A Kalman JM Aguinaga L Akar J Cabrera JA Chen SA EHRA/HRS/APHRS/SOLAECE expert consensus on atrial cardiomyopathies: definition, characterization, and clinical implication. Europace. (2016) 18:1455–90. 10.1093/europace/euw161 27402624 PMC6392440

[5] Yamaguchi T Tsuchiya T Nakahara S Fukui A Nagamoto Y Murotani K Efficacy of left atrial voltage-based catheter ablation of persistent atrial fibrillation. J Cardiovasc Electrophysiol. (2016) 27:1055–63. 10.1111/jce.13019 27235000

[6] Jadidi AS Lehrmann H Keyl C Sorrel J Markstein V Minners J Ablation of persistent atrial fibrillation targeting low-voltage areas with selective activation characteristics. Circ Arrhythm Electrophysiol. (2016) 9:e002962. 10.1161/CIRCEP.115.002962 26966286

[7] Rolf S Kircher S Arya A Eitel C Sommer P Richter S Tailored atrial substrate modification based on low-voltage areas in catheter ablation of atrial fibrillation. Circ Arrhythm Electrophysiol. (2014) 7:825–33. 10.1161/CIRCEP.113.001251 25151631

[8] Schreiber D Rieger A Moser F Kottkamp H. Catheter ablation of atrial fibrillation with box isolation of fibrotic areas: lessons on fibrosis distribution and extent, clinical characteristics, and their im- pact on long-term outcome. J Cardiovasc Electrophysiol. (2017) 28:971–83. 10.1111/jce.13278 28635186

[9] Hindricks G Potpara T Dagres N Arbelo E Bax JJ Blomström-Lundqvist C 2020 ESC guidelines for the diagnosis and management of atrial fibrillation developed in collaboration with the European Association for Cardio-Thoracic Surgery (EACTS). Eur Heart J. (2021) 42:373–498. 10.1093/eurheartj/ehaa612 32860505

[10] Jadidi A Müller-Edenborn B Chen J Keyl C Weber R Allgeier J The duration of the amplified sinus-P-wave identifies presence of left atrial low voltage substrate and predicts outcome after pulmonary vein isolation in patients with persistent atrial fibrillation. JACC Clin Electrophysiol. (2018) 4:531–43. 10.1016/j.jacep.2017.12.001 30067494

[11] Moreno-Weidmann Z Müller-Edenborn B Jadidi AS Bazan-Gelizo V Chen J Park CI Easily available ECG and echocardiographic parameters for prediction of left atrial remodeling and atrial fibrillation recurrence after pulmonary vein isolation: a multicenter study. J Cardiovasc Electrophysiol. (2021) 32:1584–93. 10.1111/jce.15013 33772926

[12] Creta A Ventrella N Providência R Earley MJ Sporton S Dhillon G Same-day discharge following catheter ablation of atrial fibrillation: a safe and cost-effective approach. J Cardiovasc Electrophysiol. (2020) 31:3097–103. 10.1111/jce.14789 33107171

[13] Creta A Kanthasamy V Schilling RJ Rosengarten J Khan F Honarbakhsh S First experience of POLARx™ versus arctic front advance™: an early technology comparison. J Cardiovasc Electrophysiol. (2021) 32:925–30. 10.1111/jce.14951 33590568

[14] Nattel S. Molecular and cellular mechanisms of atrial fibrosis in atrial fibrillation. JACC Clin Electrophysiol. (2017) 3:425–35. 10.1016/j.jacep.2017.03.002 29759598

[15] Heijman J Algalarrondo V Voigt N Melka J Wehrens XH Dobrev D The value of basic research insights into atrial fibrillation mechanisms as a guide to therapeutic innovation: a critical analysis. Cardiovasc Res. (2016) 109:467–79. 10.1093/cvr/cvv275 26705366 PMC4777910

[16] Huo Y Gaspar T Schönbauer R Wójcik M Fiedler L Roithinger FX Low-voltage myocardium-guided ablation trial of persistent atrial fibrillation. NEJM Evid. (2022) 1(11). 10.1056/EVIDoa2200141 38319851

[17] Liu HT Lee HL Wo HT Chang PC Wen MS Lin FC P wave duration ≥150 ms predicts poor left atrial function and ablation outcomes in non-paroxysmal atrial fibrillation. J Electrocardiol. (2021) 69:124–31. 10.1016/j.jelectrocard.2021.10.003 34695779

[18] Ohguchi S Inden Y Yanagisawa S Shigematsu T Yasuda K Katagiri K Long P-wave duration immediately after pulmonary vein isolation on radiofrequency catheter ablation for atrial fibrillation predicts clinical recurrence: correlation with atrial remodeling in persistent atrial fibrillation. Heart Vessels. (2022) 37(3):476–88. 10.1007/s00380-021-01932-w 34432100

[19] Providencia R Defaye P Lambiase PD Pavin D Cebron JP Halimi F Results from a multicentre comparison of cryoballoon vs. radiofrequency ablation for paroxysmal atrial fibrillation: is cryoablation more reproducible? Europace. (2017) 19:48–57. 10.1093/europace/euw027 27267554

[20] Clarnette JA Brooks AG Mahajan R Elliott AD Twomey DJ Pathak RK Outcomes of persistent and long-standing persistent atrial fibrillation ablation: a systematic review and meta-analysis. Europace. (2018) 20:366–76. 10.1093/europace/eux297 29267853

[21] Marrouche NF Wilber D Hindricks G Jais P Akoum N Marchlinski F Association of atrial tissue fibrosis identified by delayed enhancement MRI and atrial fibrillation catheter ablation: the DECAAF study. JAMA. (2014) 31:498–506. 10.1001/jama.2014.3 24496537

[22] Manios EG Kanoupakis EM Chlouverakis GI Kaleboubas MD Mavrakis HE Vardas PE. Changes in atrial electrical properties following cardioversion of chronic atrial fibrillation: relation with recurrence. Cardiovasc Res. (2000) 47(2):244–53. 10.1016/S0008-6363(00)00100-0 10946061

